# Evaluating Vitamin D Status in Pre- and Postmenopausal Type 2 Diabetics and Its Association with Glucose Homeostasis

**DOI:** 10.1155/2018/9369282

**Published:** 2018-04-02

**Authors:** Linda Ahenkorah Fondjo, Samuel Asamoah Sakyi, William K. B. A. Owiredu, Edwin Ferguson Laing, Eddie-Williams Owiredu, Ebenezer Kwesi Awusi, Richard K. D. Ephraim, Osei Sarfo Kantanka

**Affiliations:** ^1^Department of Molecular Medicine, School of Medical Sciences, College of Health Sciences, Kwame Nkrumah University of Science and Technology, Kumasi, Ghana; ^2^Department of Medical Laboratory Technology, Kwame Nkrumah University of Science and Technology, Kumasi, Ghana; ^3^Department of Medical Laboratory Technology, University of Cape Coast, Cape Coast, Ghana; ^4^Department of Medicine, Diabetic Clinic, Komfo Anokye Teaching Hospital, Kumasi, Ghana

## Abstract

**Background:**

Type 2 Diabetes Mellitus (T2DM) and menopause are associated with vitamin D status. Oestrogen decline during menopausal stages promotes hypovitaminosis D. However, the interplay between vitamin D, menopause, lifestyle, and T2DM cannot be overlooked. This study assessed vitamin D status among pre- and postmenopausal T2DM women and determined its association with glycemic control and influence of lifestyle habits on hypovitaminosis D.

**Methods:**

This cross-sectional study was conducted at the Komfo Anokye Teaching Hospital, Kumasi, Ghana. Structured questionnaires were administered to 192 T2DM women; blood samples were collected for estimation of 25(OH) D and insulin using ELISA. Fasting blood glucose (FBG), lipid profile, glycated haemoglobin (HbA1c), and calcium were measured. Statistical analyses were performed using Graphpad Prism 6.

**Results:**

The prevalence of vitamin D inadequacy was 92.2%. Hypovitaminosis D was more prevalent among the postmenopausal T2DM women (63.8% versus 58.2%). Hypovitaminosis D significantly associated with insulin [*R*^2^ = 0.01760, *p* = 0.0008], HbA1c [*R*^2^ = 0.3709, *p* = <0.0001], and FBG [*R*^2^ = 0.3465, *p* = 0.0001] in only the postmenopausal women.

**Conclusion:**

Vitamin D deficiency is prevalent in pre- and postmenopausal T2DM but higher among postmenopausal women. Adequate vitamin D levels in both groups were associated with improved glucose control while hypovitaminosis D in the postmenopausal women was related to poorer glucose control. Vitamin D screening should be incorporated into management plan for T2DM to serve as an early tool for prevention of vitamin D deficiency.

## 1. Introduction

Type 2 Diabetes Mellitus (T2DM) is a metabolic disease and major lifestyle disorder caused by either the absolute or relative insulin deficiency. T2DM is characterized by impaired glucose tolerance, chronic hyperglycemia, and altered insulin secretion [1]. It affects more than 6% of overweight urban Ghanaian adults, predominately women [2–4]. Accumulating evidence from various studies has linked vitamin D status to insulin secretion and insulin resistance [5–7]; however, the relationship between vitamin D deficiency and glycemic control remains conflicting.

Vitamin D is a steroid hormone known for its essential role in maintaining calcium homeostasis, promoting and maintaining bone health, and improving immune function [8, 9]. Vitamin D deficiency is considered a public health problem around the world. In 2008, it was estimated that 1 billion persons present with vitamin D insufficiency or deficiency [10]. Vitamin D is obtained through exposure to ultraviolet B (UVB) sunlight as well as nutritional sources. Despite the high UVB sunlight exposure in tropical countries, studies suggest vitamin D deficiency is prevalent and is further influenced by age and gender [11, 12].

Menopause, the cessation of menstrual cycle caused by reduced secretions of estrogen and progesterone, is defined as 1 year without menses, occurring between the ages of 45–55 [13]. Oestrogen increases the activity of 1-*α*-hydroxylase (expressed in the kidneys) responsible for the activation of vitamin D and upregulates the vitamin D receptor (VDR) [14]. During menopausal stages, there is a gradual reduction in amount of oestrogen produced by the ovaries [15]; this decline in oestrogen production is thought to promote vitamin D deficiency. The ensuing vitamin D challenge is related to decrease in number of vitamin D receptors [14, 16, 17].

Aging in women and the subsequent drop in oestrogen levels are thus associated with decline in vitamin D levels. We have previously established a high prevalence of vitamin D deficiency among diabetics and nondiabetics in Ghana [4]. The interplay between sunlight exposure, lifestyle habits, and serum vitamin D levels cannot be disregarded. Besides, these two factors may not be substantive enough to prevent hypovitaminosis D among Ghanaian women with T2DM. We postulate that age and lifestyle habits are associated with reduction in vitamin D levels and a negative association exists between vitamin D deficiency and glycemic control. This study therefore evaluated the levels of 25(OH) vitamin D in pre- and postmenopausal T2DM women and assessed the association between vitamin D deficiency and glycemic control as well as the influence of lifestyle habits on the development of vitamin D deficiency.

## 2. Methodology

### 2.1. Study Design

This cross-sectional study was conducted at the Diabetic Clinic of the Komfo Anokye Teaching Hospital (KATH), Kumasi, Ghana, between January and March 2017.

### 2.2. Study Site

The study was conducted at Komfo Anokye Teaching Hospital (KATH) in Kumasi, Ghana, West Africa. KATH is the second largest hospital in Ghana and it is located in the sublocality, Kumasi in the Kumasi Metropolitan District, Ashanti Region of Ghana. It lies between latitude 6.35°N and 6.40°N and longitude 1.3°W and 1.35°W. It is the second major city in Ghana with a population of 1.2 million [18]. The diabetic clinic is visited by more than 100 patients per week [19].

### 2.3. Ethical Considerations

Ethical approval for this study was obtained from the Committee on Human Research Publication and Ethics (CHRPE) of the School of Medical Sciences, Kwame Nkrumah University of Science and Technology (CHRPE/AP/084/17) as well as from the Research and Development Department of KATH and the management of the Diabetic Clinic, KATH. All participants gave their written informed consent after the aim and objectives of the study had been explained to them.

### 2.4. Study Population

The sample size for the study was calculated using Fischer's sampling formula (*N* = *Z*^2^*PQ*/*d*^2^), where *Z* is the critical value of the normal distribution (1.96 at 95% CI); *P* is the estimated prevalence of T2DM in Ghana (6%); *Q* = 100 − *P*; and *d* is the absolute precision or sampling error tolerated = 5%. From the above equation, a total of 192 Ghanaian women, 98 premenopausal and 94 postmenopausal clinically diagnosed with T2DM, living in Kumasi were recruited for the study. All respondents attended the Diabetic Clinic of the Komfo Anokye Teaching Hospital and had had diabetes for more than 6 months.

### 2.5. Questionnaire Administration

Structured questionnaires were administered to obtain sociodemographic and medical history of study participants. The questionnaires were designed by reviewing previous studies of similar objective and were tailored to fit our study objectives (face validity). The questionnaire was pilot tested; the data was entered into excel sheet and cleaned before it was administered to the study participants.

### 2.6. Inclusion and Exclusion Criteria

Clinically diagnosed T2DM patients, 25 years and above, of more than six months' duration were included in the study. Respondents with osteoporosis, cancer, renal failure (renal osteodystrophy), and liver disease and those on medication that could affect glucose or lipid metabolism, vitamin D metabolism or its absorption (phenytoin, rifampin, isoniazid, and ketoconazole), and calcium supplementation were excluded from the study.

### 2.7. Blood Pressure Measurement

Blood pressure was measured with an automated blood pressure apparatus (Omron MX3-Omron Matsusaka Co., Ltd. Japan) from the right arm after the subjects had been sitting for about five minutes. The average of the two readings taken five minutes apart was recorded as the blood pressure measurement. Hypertension was defined as a systolic blood pressure ≥ 140 mm Hg or diastolic blood pressure ≥ 90 mm Hg or history of previously known disease [20].

### 2.8. Anthropometric Evaluation

The weight of the selected subjects was measured in light clothing without shoes, in an upright position to using a calibrated analogue scale (Seca, Hamburg, Deutschland) (the nearest 0.1 kg). Height was measured without shoes using a stadiometer (Seca, Hamburg, Deutschland) (to the nearest 0.1 cm). Waist circumference (WC) and hip circumference (HC) were measured (to the nearest 0.1 cm) with a measuring tape. All anthropometric measurements were carried out on all respondents by same trained personnel. Body mass index (BMI) was calculated using the equation; [BMI (kg/m^2^) = weight/height^2^]. Waist to hip ratio (WHR) was calculated as waist circumference divided by hip circumference and waist to height ratio (WHtR) was calculated as waist circumference divided by height. Visceral adiposity index (VAI) and body adiposity index (BAI) were calculated using the formulae below:

For females,

(1) VAI=WC36.58+1.89×BMI×TG0.81×1.52HDL,BAI=hip circumferenceheight in m×√height−18.

### 2.9. Sample Collection and Preparation

Ten (10) milliliters of blood was collected from the antecubital vein of each respondent between the hours of 8 am to 11 am after an overnight fast for the biochemical assays. Two (2) milliliters of blood was dispensed into tubes containing fluoride oxalate, another 2 milliliters was dispensed into tubes containing EDTA, and the remaining 6 ml of blood was dispensed into gel separator tubes. The tubes were placed in a centrifuge and spun at 3000 rpm for 10 minutes to obtain the plasma and serum. Plasma glucose was measured immediately and the serum and plasma for the measurement of other biochemical variables were stored at −20°C until analysis.

### 2.10. Biochemical Assays

The biochemical reagent for the determination of serum vitamin D (25(OH) D) and insulin of the study participants were purchased from Biobase Biodustry (Shandong) Co., Ltd., China, and analyzed based on the principle of sandwich Enzyme Linked Immunosorbent Assay, using a polystyrene microtiter plate (Biobase Biodustry (Shandong) Co., Ltd., China) according to the manufacturer's instructions. The absorbance of the colour produced was measured spectrophotometrically at 450 nm using Thermo Electron Multiskan EX plate reader (Shanghai, China). Serum vitamin D level was stratified into normal (≥30 ng/ml), insufficient (≥20 to <30 ng/ml), and deficient (<20 ng/ml) [9, 14]. The reagents used to determine calcium, fasting blood glucose, total cholesterol, high density lipoprotein, triglyceride, and glycated haemoglobin levels were purchased from Biosystem, Barcelona, Spain, and estimated enzymatically with a chemistry analyzer (Biosystem A25, Barcelona, Spain). The absorbance of each analyte was determined spectrophotometrically at wavelength of 505 nm. Low density lipoprotein (LDL) cholesterol concentration was determined using Friedewald's formula: LDL cholesterol (mmol/L) = total cholesterol (mmol/L) – HDL cholesterol (mmol/L) – [triglyceride (mmol/L)/2.2] [21].

The Homeostasis Model Assessment (HOMA) was used to determine insulin resistance (HOMA-IR) and beta cell function (insulin secretory capacity) (HOMA-*β*) using the calculated formulae [22]:

(2) HOMA-IR=fasting insulin IU/ml×fasting blood glucose22.5,HOMA-β=20×fasting insulin IU/mlfasting blood glucose−3.5%.

### 2.11. Statistical Analysis

All data were presented as frequency (percentages) and mean ± SD. Student's *t*-test and Chi-square test statistic were used to test association between variables. Linear regression analysis was performed to test association between biochemical parameters. Binary logistic regression analysis was also performed to determine the odds of sociodemographic, lifestyle, and anthropometric indices in predicting the development of vitamin D deficiency. In the binary logistic regression analysis, age was adjusted to correct the differences in ages among the pre- and postmenopausal women. A *p* value < 0.05 was considered statistically significant. All statistical analyses were performed using IBM SPSS 21.0 Statistics and Graphpad Prism 6 version 6.01.

## 3. Results

In this study, of the 192 T2DM women recruited, 98 (51.0%) were premenopausal while 94 (49.0%) were postmenopausal. A higher proportion of the participants were within age range 41–50 years (47.4%) and were married (62.0%). Majority of the participants had basic education (51.6%), were employed (63.0%), and were Christians (89.6%). Smoking was self-reported and none of the participants had a history of smoking, while 3.1% consumed alcohol. Majority of women (39.6%) had been diagnosed with T2DM between 6 and 10 years. Furthermore, 50% of the participants had family history of diabetes (Table 1).

There was a higher proportion of overweight patients (36.5%) based on their BMI. Majority of the participants (89.6% and 66.7%) were obese based on WHR and WHtR, respectively. Moreover, most of the patients were prehypertensive (34.9%). However, a higher percentage of the postmenopausal women were hypertensive (Stages 1 and 2) (Table 2).

**Figure 1:**
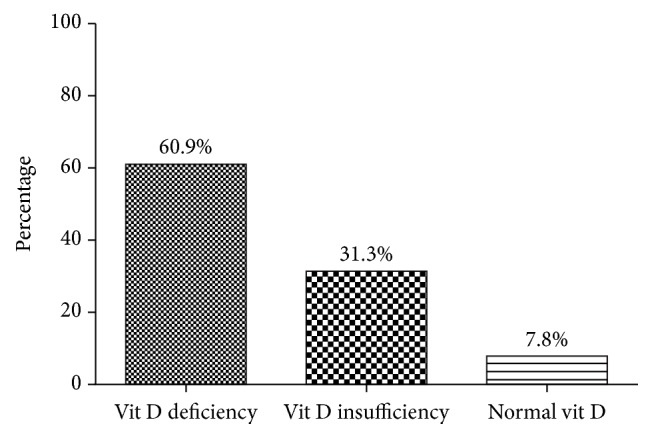
Vitamin D status of the entire study population.

The prevalence of vitamin D deficiency (<20 ng/ml), vitamin D insufficiency (≥20 ng/ml to <30 ng/ml), and normal vitamin D (>30 ng/ml) was 60.9%, 31.3%, and 7.8%, respectively (Figure 1).

**Figure 2:**
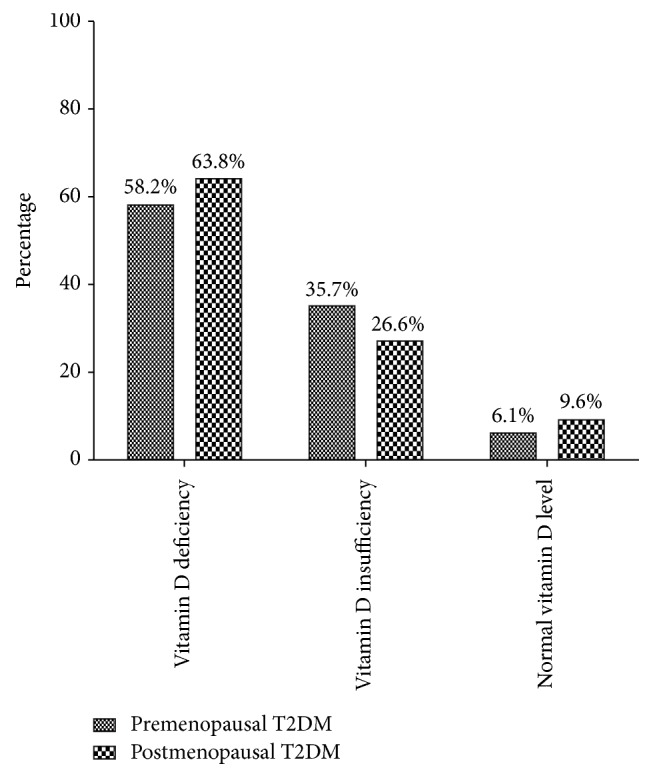
Vitamin D status among pre- and postmenopausal T2DM women.

The prevalence of vitamin D deficiency, vitamin D insufficiency, and normal vitamin D observed among premenopausal T2DM women was 58.2%, 35.7%, and 6.1%, respectively. Furthermore, 63.8% of postmenopausal T2DM women presented with vitamin D deficiency, 26.6% were vitamin D insufficient, and 9.6% had normal vitamin D levels. Vitamin D deficiency was more prevalent in postmenopausal T2DM women (Figure 2).

Vitamin D levels and HOMA-IR were significantly reduced among postmenopausal T2DM women compared to premenopausal participants (*p* = 0.0002; *p* value < 0.0001). There was a significantly elevated systolic blood pressure among postmenopausal T2DM women (*p* = 0.011). In both categories of women, there were obesity, elevated blood glucose levels with poor glycemic control, and deranged lipid metabolism, with the postmenopausal women presenting with significantly elevated total cholesterol (*p* = 0.044) and fasting insulin (<0.0001) (Table 3).

There were significant elevated FBG, HbA1c, and HOMA-IR in postmenopausal vitamin D deficient groups compared to nondeficient participants, with a corresponding significant decrease in insulin and vitamin D. With the exception of TCHOL, HDL-C, and vitamin D, there was no significant difference between vitamin D deficient and nondeficient premenopausal participants as shown in Table 4. 

Significantly greater number of postmenopausal vitamin D deficient participants were hyperglycemic (98.3%) compared to nondeficient participants (55.9%) (*p* value < 0.001). Moreover, a significant percentage (31.7%) of the postmenopausal vitamin D deficient participants had a very poor glucose control (HbA1c) compared to nondeficient patients (2.9%) while most of the nondeficient patients had a good glucose control (82.4%) (*p* value < 0.001). Similarly, the postmenopausal vitamin D deficient women presented with reduced beta cell function (Table 4).

With the exception of the hemodynamic profile of the vitamin D deficient postmenopausal women, all parameters showed no statistically significant difference. However, there was elevated WHR among both pre- and postmenopausal vitamin D deficient subjects compared to nondeficient subjects. Premenopausal vitamin D deficient subjects had elevated systolic pressure, BMI, and WHtR, when compared to nondeficient subjects, contrary to postmenopausal vitamin D deficient subjects who had reduced systolic pressure, BMI, and WHtR. Similarly, obesity was widespread among the vitamin D deficient women in both groups (Table 5).

Vitamin D deficiency did not show any statistical significant association with FBG, HbA1c, fasting insulin, HOMA-IR, and HOMA-*β* among premenopausal T2DM women.

However, there was a significant negative association vitamin D sufficiency in the premenopausal T2DM women and HbA1c [*r*^2^ = 0.1322, *p* value = 0.0195] and a borderline significance for FBG [*r*^2^ = 0.3140, *p* value = 0.0579] (Table 6).

There was statistically significant inverse association between vitamin D level and fasting blood glucose and with HbA1c among both vitamin D deficient and nondeficient women but there was a positive association between vitamin D deficiency and fasting insulin. Conversely, there was no statistically significant association between vitamin D and HOMA-*β* and also HOMA-IR among both vitamin D deficient and nondeficient T2DM women (Table 6).

Increasing age among menopausal T2DM women and higher BMI (cOR = 0.763, 95% CI (0.417–1.397), *p* = 0.381) did not influence the development of vitamin D deficiency. However, though not statistically significant, a greater risk of developing vitamin D deficiency was associated with unemployment (cOR = 1.573, 95% CI (0.851–2.905), *p* = 0.148), being both uneducated (cOR 1.194, 95% CI (0.105–13.622), *p* = 0.105) and educated (2.387, 95% CI (0.257–22.173), *p* = 0.257), having diabetes mellitus for >5 years (cOR = 1.824, 95% CI (0.942–3.531), *p* = 0.075), higher WHR (cOR = 1.375, 95% CI (0.581–3.253), *p* = 0.469), and WHtR (OR = 1.340, 95% CI (0.728–2.469), *p* = 0.347) (Table 7).

Upon adjusting for age, similar results were observed. Higher BMI (aOR = 0.749, 95% CI (0.408–1.376), *p* = 0.352) did not influence the development of vitamin D deficiency. Again, a higher risk of developing vitamin D deficiency was associated with unemployment (aOR = 1.612, 95% CI (0.828–3.138), *p* = 0.160), being both uneducated (1.095, 95% CI (0.094–12.800), *p* = 0.943) and educated (2.236, 95% CI (0.222–22.529), *p* = 0.495), having diabetes mellitus for >5 years (aOR = 1.842, 95% CI (0.926–3.664), *p* = 0.082), higher WHR (aOR = 1.419, 95% CI (0.594–3.392), *p* = 0.431), and WHtR (aOR = 1.336, 95% CI (0.723–2.468), *p* = 0.355) (Table 7).

## 4. Discussion

Vitamin D deficiency among diabetics has been described in different population with varying prevalence. Given the higher prevalence of diabetic population among females in Ghana, this study determined the status of vitamin D in pre- and postmenopausal diabetic women and assessed the relationship between vitamin D deficiency and markers of glycemic control and the influence of lifestyle on the development of vitamin D deficiency.

This study observed that 92.2% (deficiency and insufficiency) of the diabetic women studied presented with vitamin D inadequacies regardless of the relative abundance of sunshine in Ghana. Additionally, it is noteworthy that hypovitaminosis D was more widespread in the postmenopausal T2DM women (63.8% versus 58.2%) from our study (Figures 1 and 2).

In a cross-sectional study in Japan, Mori and colleagues indicated that 91.8% of postmenopausal diabetic women are deficient of vitamin D [23]. In another cross-sectional study in India, Kanwar and coworkers reported a higher prevalence of vitamin D deficiency among postmenopausal T2DM women compared to premenopausal T2DM women (80% versus 60%) [14]. Another cross-sectional study in Indonesia by Hidayat et al. observed a prevalence of 78.2% vitamin D deficiency among elderly T2DM women [24]. Likewise, studies by Sarmidi et al. [25] and Setiati and Sutrisna [26] observed a prevalence of 61.9% and 35.1%, respectively.

The marginally lower prevalence in Setiati's study compared to our present study (35.1% versus 60.9%) could be related to the fact that the study participants were elderly females who lived in institutional care homes, where a regular medication schedule, which may include multivitamins (calcium and vitamin D), a balanced dietary patterns, and possibly the timed sunlight exposure, may account for the high disparity.

Vitamin D synthesis by the skin is thought to represent the major source of vitamin D; however, the finding of high vitamin D deficiency among the study participants emphasizes our earlier predisposition that vitamin D status is not mainly dependent on sunshine exposure, but dietary and underlying health conditions play a huge role especially in the Ghanaian population where sunshine is in abundance [4]. Moreover, in this present study, more women were either overweight or obese. Additionally, the vitamin D deficient women had unhealthy weight profiles (Tables 2 and 5). Findings by Fondjo and colleagues [4], Rodríguez-Rodríguez et al. [27], and Taheri et al. [28] on obesity and vitamin D are in keeping with our current study.

Obesity increases the risk for hypovitaminosis D due to deposition of vitamin D precursors in body fat stores, reducing its bioavailability to the skin [8, 29, 30]. It is noteworthy that most of our respondents had attained only basic education (Table 1). Undeniably, among the uneducated and semiliterate Ghanaians, obesity is associated with a sign of affluence and hence these women make a conscious effort to gain weight in order to be perceived as leading a good life or being adequately taken care of by their spouse or partner. Additionally, level of education from our study influenced the development of vitamin D deficiency of the participants (Table 7). This could possibly account for the high prevalence of vitamin D deficiency in our study participants.

The finding of a higher vitamin D deficiency in the postmenopausal women may be related advanced age. Aging is directly related to decreasing vitamin D levels. The diminishing levels of 25(OH) D with age is due to impaired intestinal absorption of vitamin D [31] as well as a decline in the concentration of vitamin D precursors normally stored in the skin coupled with reduced capacity to synthesize vitamin D in the skin when exposed to UVB radiation [32]. Additionally, decline in oestrogen associated with postmenopausal women decreases the activity of 1-alpha hydroxylase vitamin D responsible for activating vitamin D and its receptors (VDRs).

The effects of poor metabolic control on 25(OH) vitamin D levels have been identified by other studies, but remain largely unpredictable. In our earlier study in Ghana, we reported no significant association between vitamin D deficiency and HOMA-IR as well as HOMA-*β* among diabetic cases and nondiabetic controls. This current study did not find an association between vitamin D deficiency and HOMA-IR and HOMA-*β* in both pre- and postmenopausal T2DM women (Figure 2 and Table 6). However, vitamin D deficiency significantly associated with FBG, HbA1c, and insulin in the postmenopausal women. This in part corroborates our earlier findings on vitamin D deficiency and HOMA-IR and HOMA-*β* [4].

Need and colleagues as well as Ford et al.'s study [33] reported an inverse relationship between FBG and serum 25(OH) D levels. A cross-sectional study by Doddamani et al. on newly detected type 2 diabetics also reported an inverse association between vitamin D and FBG. They also reported higher HbA1c levels in patients with severe vitamin D deficiency compared to subjects with mild to moderate deficiency [34]. Similarly, Dalgård et al. [35] and Shanthi et al. [36] observed an inverse association between vitamin D and HbA1c. Chiu and colleagues in a cross-sectional study also observed a negative correlation between serum 25(OH) D_3_ and postprandial glucose concentration and a positive association between vitamin D and insulin [37]. The observed association between vitamin D, FBG, and HBA1c (Table 6) is suggestive of the fact that good control of blood sugar is essential for optimal vitamin D levels among diabetic women. The finding of a significant negative association between vitamin D sufficiency and FBG and HBA1c in both pre- and postmenopausal women further corroborates this assertion. Moreover, a greater number of the vitamin D deficient study participants presented with poorer glycemic control and a higher blood glucose levels (Table 4). Pannu et al., 2017, in a population based study in Australia have reported on a protective effect of higher 25(OH) D on FPG and HbA1c [38].

Vitamin D enhances insulin production by the pancreatic *β*-cells [39] and appropriate amount of insulin regulates blood glucose to optimal levels thereby reducing the rate of haemoglobin glycosylation. This inverse association may be because vitamin D improves insulin exocytosis via activating calcium-dependent endopeptidases, hence, accounting for the high insulin levels in the presence of high vitamin D levels [39] (Table 4). Therefore, adequate vitamin D levels may lead to reduction in the blood glucose level through adequate insulin production.

Our findings also suggest that although vitamin D correlates with blood glucose levels, insulin, and overall glucose maintenance, its association with improving insulin resistance and pancreatic beta function requires additional interventional studies. Moreover, Witham et al. [40], Kampmann et al. [41], and Elkassaby et al. [42] in a double-blind, randomized, placebo-controlled trial reported that improvement in vitamin D status had no effect on insulin resistance and beta cell function in T2DM patients. Furthermore, an intervention studies by Borissova et al. among Bulgarian women with T2DM showed that there was no significant decrease in HOMA-IR upon one-month oral, 1332 IU cholecalciferol/day [43].

Although Inzucchi et al. [44] observed improvement in insulin sensitivity of respondents on supplementation, improvements were observed in study participants whose vitamin D levels had increased from 10 to 30 ng/ml.

BMI was elevated in both groups however; high BMI is not associated with increased risk of vitamin D deficiency among our study participants (Table 7). This could be attributed to the fact that BMI is a poor obesity index as compared to WHtR and WHR [45]. Increased WHR, WHtR, having diabetes mellitus for >5 years, educational level, and unemployment were independent risk factors for vitamin D deficiency in this study (Table 7). Unemployment could lead to financial insecurities as such resulting in poorer nutritional choices while the possibility of developing diabetic nephropathy, which could affect the function of 1-alpha hydroxylase (expressed predominantly in the kidney) required in the activation of vitamin D, could account for the observed risk factors.

Although hypovitaminosis D frequently occurs without symptoms, apart from regulating glycemic index, it poses a risk for osteoporosis and hip fractures [46] in females. Thus, screening for vitamin D deficiency among diabetics and obese is encouraged.

This study is limited by the fact that it was cross-sectional with inferences made from a group rather than individuals. The causality of T2DM in the pre- and postmenopausal women cannot be established. Oestrogen was not estimated and dietary recall was not possible among the diabetic women in this study; however this does not have effect on the aims and findings of this study but contributes to scientific knowledge.

## 5. Conclusion

Vitamin D deficiency is high in both pre- and postmenopausal T2DM especially among postmenopausal T2DM women. Vitamin D adequacy in both groups improves overall glucose control while vitamin D deficiency in the postmenopausal women is associated with poor glucose control. Obesity, unemployment, and diabetes mellitus more than five years are independent risk factors for developing vitamin D deficiency. Adequate vitamin D may play a role in long term glucose control. Vitamin D screening and supplementation should be incorporated into management plan for all T2DM women to serve as an early tool for prevention of vitamin D deficiency.

## Figures and Tables

**Table 1:** Sociodemographic and lifestyle characteristics of study participants.

Variables	Total (*n* = 192)	Premenopausal (*n* = 98)	Postmenopausal (*n* = 94)	*p* value
Mean age (years)		43.54	68.70	
Age range				
39-40	7 (3.6)	7 (7.1)	0 (0.0)	**<0.0001**
41–50	91 (47.4)	91 (92.9)	0 (0.0)	
51–60	17 (8.9)	0 (0.0)	17 (18.1)	
61–70	77 (40.1)	0 (0.0)	77 (81.9)	
Marital status				
Single	73 (38.0)	25 (25.5)	48 (51.1)	**0.0003**
Married	119 (62.0)	73 (74.5)	46 (48.9)	
Educational level				
Basic	99 (51.6)	49 (50.0)	50 (53.2)	**0.0193**
Secondary	85 (44.2)	49 (50.0)	36 (38.3)	
Tertiary	5 (2.6)	0 (0.0)	5 (5.3)	
Illiterate	3 (1.6)	0 (0.0)	3 (3.2)	
Employment status				
Employed	121 (63.0)	76 (77.5)	45 (47.9)	**<0.0001**
Unemployed	71 (37.0)	22 (22.4)	49 (52.1)	
Religion				
Christian	172 (89.6)	87 (88.8)	85 (90.4)	0.3741
Muslim	20 (10.4)	11 (11.2)	9 (9.6)	
Lifestyle habits				
Alcohol intake				
Yes	6 (3.1)	5 (5.1)	1 (1.1)	0.608
No	186 (96.9)	93 (94.9)	93 (98.9)	
Family history of DM				**0.0001**
Yes	96 (50.0)	27 (27.6)	69 (73.4)	
No	96 (50.0)	71 (72.4)	25 (26.6)	
Duration of DM				
<1 year	10 (5.2)	7 (7.1)	3 (3.2)	**<0.0001**
1–5 years	38 (19.8)	27 (27.6)	11 (11.7)	
6–10 years	76 (39.6)	64 (65.3)	12 (12.8)	
>10 years	68 (35.4)	0 (0)	68 (72.3)	

Chi square analysis was performed to compare categorical variables among pre- and postmenopausal T2DM women. Values are presented as mean ± SD. *p* < 0.05 was considered statistically significant (*p* values of significant variables are in bold print). *χ*^2^: Chi square; df: degree of freedom.

**Table 2:** Anthropometric and hemodynamic characteristics of study participants.

Variables	Total (*n* = 192)	Premenopausal (*n* = 98)	Postmenopausal (*n* = 94)	*p* value
BMI categories				
Underweight	4 (2.1)	0 (0.0)	4 (4.2)	0.1991
Healthy	51 (26.6)	28 (28.6)	23 (24.5)	
Overweight	70 (36.5)	37 (37.8)	33 (35.1)	
Obese	67 (34.8)	33 (33.7)	34 (36.2)	
WHR categories				
Healthy	5 (2.6)	3 (3.1)	2 (2.1)	0.8709
Overweight	15 (7.8)	7 (7.1)	8 (8.5)	
Obese	172 (89.6)	88 (89.8)	84 (89.4)	
WHtR categories				
Healthy	7 (3.6)	3 (3.1)	4 (4.3)	0.7688
Overweight	20 (10.4)	12 (12.2)	8 (8.5)	
Very overweight	37 (19.3)	20 (20.4)	17 (18.1)	
Obese	128 (66.7)	63 (64.3)	65 (69.1)	
HTN categories				
Normotension	45 (23.4)	31 (31.6)	14 (14.9)	**0.0374**
Pre-HTN	67 (34.9)	32 (32.7)	35 (37.2)	
Stage 1	53 (27.6)	25 (25.5)	28 (29.8)	
Stage 2	27 (14.1)	10 (10.2)	17 (18.1)	

Chi square analysis was performed to compare categorical variables among pre- and postmenopausal T2DM women. Values are presented as mean ± SD. *p* < 0.05 was considered statistically significant (*p* values of significant variables are in bold print). *χ*^2^: Chi square; df: degree of freedom; BMI: body mass index; WHR: waist to hip ratio; WHtR: waist to height ratio; HTN: hypertension; Normotension: systolic pressure (<120 mmHg); Pre-HTN: systolic pressure (120–139 mmHg); Stage 1: systolic pressure (140–159 mmHg); Stage 2: systolic pressure (>160 mmHg).

**Table 3:** Biochemical, hemodynamic, and anthropometric characteristics of the study participants stratified by stage of menopause.

Variables	Premenopausal	Postmenopausal	*p* value
Biochemical parameters			
FBG (mmol/L)	10.5 ± 5.03	9.60 ± 4.34	0.187
TCHOL (mmol/L)	4.58 ± 1.30	4.96 ± 1.29	**0.044**
TG (mmol/L)	1.22 ± 0.60	1.36 ± 0.76	0.157
HDL-C (mmol/L)	1.28 ± 0.47	1.29 ± 0.39	0.873
LDL-C (mmol/L)	2.75 ± 1.00	3.05 ± 1.16	0.056
HbA1c (%)	8.42 ± 2.77	7.71 ± 2.24	0.053
Calcium (mmol/L)	2.18 ± 0.3	2.17 ± 0.25	0.911
Fasting insulin (mU/L)	15.85 ± 4.73	20.45± 4.87	**<0.0001**
Vitamin D (nmol/L)	22.08 ± 5.98	19.73 ± 6.05	**0.0002**
HOMA-IR	6.98 ± 3.01	3.03 ± 0.64	**<0.0001**
HOMA-*β*	103.46 ± 188.65	82.66 ± 52.91	0.304
Anthropometry			
Height (m)	1.59 ± 0.63	1.59 ± 0.08	1.000
Weight (kg)	71.83 ± 13.85	70.67 ± 13.85	0.563
BMI (kg/m^2^)	28.32 ± 5.07	28.13 ± 5.54	0.804
WC (m)	0.97 ± 0.15	0.98 ± 0.13	0.623
HC (m)	1.04 ± 0.10	1.05 ± 0.13	0.550
WHR	0.94 ± 0.14	0.94 ± 0.08	1.000
WHtR	0.61 ± 0.09	0.62 ± 0.09	0.286
BAI	33.89 ± 5.41	34.69 ± 7.61	0.401
VAI	2.24 ± 1.50	2.50 ± 2.05	0.316
Hemodynamic profile			
Systole	131.52 ± 22.39	140.01 ± 23.14	**0.011**
Diastole	79.89 ± 13.30	78.89 ± 12.01	0.586
MAP	97.20 ± 15.23	99.27 ± 14.24	0.332

Continuous variables are presented as mean ± standard deviation (SD). Students' *t*-test analysis was performed to compare means of pre- and postmenopausal T2DM women. *p* < 0.05 was considered statistically significant (*p* values of significant variables are in bold print). FBG: fasting blood glucose; TCHOL: total cholesterol; TG: triglycerides; HDL-C: high density lipoprotein cholesterol; LDL-C: low density lipoprotein cholesterol, HbA1C: glycated haemoglobin; Calcium: total calcium; HOMA-IR: Homeostasis Model Assessment-Insulin Resistance; HOMA-*β*: Homeostasis Model Assessment-*β*; BMI: body mass index; WC: waist circumference; HC: hip circumference; WHR: waist to hip ratio; WHtR: waist to height ratio; BAI: body adiposity index; VAI: visceral adiposity index; MAP: mean arterial pressure.

**Table 4:** Biochemical profile of study groups stratified by vitamin D status.

Variables	Premenopausal T2DM			Postmenopausal T2DM		
	Vitamin D deficient (<20 ng/ml), *n* = 57	Vitamin D sufficient (>20 ng/ml), *n* = 41		Vitamin D deficient (<20 ng/ml), *n* = 60	Vitamin D sufficient (>20 ng/ml), *n* = 34	
Biochemical parameters			*p* value			*p* value

FBG	11.14 ± 5.14	9.6 ± 4.79	0.136	11.39 ± 4.49	6.63 ± 1.43	**<0.0001**
Normoglycemia	13 (22.8)	10 (24.4)	0.8552	1 (1.7)	15 (44.1)	**<0.0001**
Hyperglycemia	44 (77.2)	31 (75.6)		59 (98.3)	19 (55.9)	
TCHOL	4.39 ± 1.07	4.83 ± 1.53	**0.0097**	4.92 ± 1.41	4.98 ± 1.05	0.818
Low	18 (31.6)	13 (31.7)	0.8449	16 (26.7)	6 (17.6)	0.4173
Normal	25 (43.9)	15 (36.6)		19 (31.7)	15 (44.1)	
High	14 (24.6)	13 (31.7)		25 (41.7)	13 (38.2)	
TG	1.19 ± 0.59	1.26 ± 0.41	0.515	1.44 ± 0.90	1.22 ± 0.35	0.141
Low	5 (8.8)	2 (4.9)	0.7186	2 (3.3)	0 (0.0)	0.1173
Normal	50 (87.7)	38 (92.7)		53 (88.3)	34 (100.0)	
High	2 (3.5)	1 (2.4)		5 (8.3)	0 (0.0)	
HDL-C	1.24 ± 0.49	1.33 ± 0.44	**0.0352**	1.27 ± 0.41	1.33 ± 0.36	0.454
Low	10 (17.5)	7 (17.1)	0.7780	10 (16.7)	3 (8.8)	0.298
Normal	44 (77.2)	33 (80.5)		48 (80.0)	31 (91.2)	
High	3 (5.3)	1 (2.4)		2 (3.3)	0 (0.0)	
LDL-C	2.61 ± 0.84	2.93 ± 1.18	0.120	3.02 ± 1.24	3.10 ± 1.01	0.735
Normal	47 (82.5)	28 (68.3)	0.1027	47 (78.3)	25 (73.5)	0.5286
High	10 (17.5)	13 (31.7)		13 (21.7)	9 (26.5)	
HbA1c	8.63 ± 2.60	8.12 ± 2.99	0.371	8.48 ± 2.36	6.35 ± 1.06	**<0.0001**
Good	17 (29.8)	16 (39.0)	0.5907	10 (16.7)	28 (82.4)	**<0.0001**
Fair	11 (19.3)	8 (19.5)		21 (35.0)	4 (11.8)	
Poor	8 (14.0)	7 (17.1)		10 (16.7)	1 (2.9)	
Very poor	21 (36.8)	10 (24.4)		19 (31.7)	1 (2.9)	
Calcium	2.11 ± 0.27	2.23 ± 0.32	0.050	2.17 ± 0.27	2.19 ± 0.22	0.697
Low	21 (36.8)	16 (39.0)	0.9751	30 (50.0)	20 (58.8)	0.6005
Normal	33 (57.9)	23 (56.1)		26 (43.3)	13 (38.2)	
High	3 (5.3)	2 (4.9)		4 (6.7)	1 (2.9)	
Insulin	15.25 ± 4.28	16.68 ± 5.22	0.140	19.75 ± 4.39	21.68 ± 5.47	0.056
Vitamin D	16.16 ± 2.56	24.94 ± 4.55	**<0.0001**	16.08 ± 3.25	26.16 ± 4.19	**<0.0001**
HOMA-IR	7.03 ± 2.68	6.91 ± 3.436	0.847	3.11 ± 0.58	2.91 ± 0.75	0.140
HOMA-*β*	82.62 ± 92.82	132.42 ± 269.75	0.099	56.03 ± 27.58	124.64 ± 54.84	**<0.0001**

Continuous variables are presented as mean ± standard deviation (SD). Students' *t*-test analysis was performed to compare means of continuous variables. Categorical variables were expressed as frequency (percentage). Differences between categorical variables were analyzed with Chi square analysis. *p* < 0.05 was considered statistically significant (*p* values of significant variables are in bold print) FBG: fasting blood glucose; TCHOL: total cholesterol; TG: triglycerides; HDL-C: high density lipoprotein cholesterol; LDL-C: low density lipoprotein cholesterol; HbA1C: glycated haemoglobin; Calcium: total calcium; HOMA-IR: Homeostasis Model Assessment-Insulin Resistance; HOMA-*β*: Homeostasis Model Assessment-*β*eta.

**Table 5:** Hemodynamic and anthropometric characteristics of study groups stratified by vitamin D status.

Variables	Premenopausal T2DM			Postmenopausal T2DM		
	Vitamin D deficient	Vitamin D sufficient		Vitamin D deficient	Vitamin D sufficient	
	(<20 ng/ml) *n* = 57	(>20 ng/ml) *n* = 41		(<20 ng/ml) *n* = 60	(>20 ng/ml) *n* = 34	
Hemodynamic profile			*p*-value			*p*-value
Systole	133.81 ± 21.73	128.32 ± 23.16	0.233	135.82 ± 21.96	147.41 ± 23.62	**0.019**
Diastole	79.18 ± 11.49	80.88 ± 15.55	0.535	76.75 ± 11.46	82.68 ± 12.38	**0.021**
MAP	97.38 ± 13.51	96.69 ± 17.50	0.826	96.44 ± 13.48	104.25 ± 14.39	**0.001**
Anthropometrics						
Weight	71.45 ± 14.51	72.36 ± 13.02	0.750	69.37 ± 14.21	72.96 ± 13.09	0.238
BMI	28.39 ± 5.43	28.21 ± 4.57	0.863	27.68 ± 5.30	28.95 ± 5.93	0.288
Underweight				3 (5.0)	1 (2.9)	0.253
Healthy	17 (29.8)	11 (26.8)	0.913	14 (23.3)	9 (26.5)	
Overweight	20 (35.1)	16 (39.0)		25 (41.7)	8 (23.5)	
Obese	20 (35.1)	14 (34.2)		18 (30.0)	16 (47.1)	
WHR	0.94 ± 0.16	0.93 ± 0.08	0.714	0.94 ± 0.08	0.93 ± 0.08	0.562
Healthy	1 (1.8)	2 (4.9)	0.651	1 (1.7)	2 (5.9)	0.353
Overweight	2 (3.5)	1 (2.4)		4 (6.7)	4 (11.8)	
Obese	54 (94.7)	38 (92.7)		55 (91.7)	28 (82.4)	
WHtR	0.62 ± 0.16	0.59 ± 0.66	0.742	0.61 ± 0.09	0.63 ± 0.09	0.303
Healthy	1 (1.8)	2 (4.9)	0.438	4 (6.7)	1 (2.9)	0.101
Overweight	9 (15.8)	3 (7.3)		6 (10.0)	2 (5.9)	
Very overweight	10 (17.5)	10 (24.4)		6 (10.0)	10 (29.4)	
Morbidly obese	37 (64.9)	26 (63.4)		44 (73.3)	21 (61.8)	

Continuous variables are presented as mean ± standard deviation (SD). Student's *t*-test analysis was performed to compare means of continuous variables. Categorical variables were expressed as frequency (percentage). Differences between categorical variables were analyzed with Chi square analysis. *p* < 0.05 was considered statistically significant (*p* values of significant variables are in bold print). BMI: body mass index; WC: waist circumference; HC: hip circumference; WHR: waist to hip ratio; WHtR: waist to height ratio; BAI: body adiposity index; VAI: visceral adiposity index; MAP: mean arterial pressure.

**Table 6:** Association between vitamin D status and glycemic indices in premenopausal and postmenopausal T2DM women.

	Premenopausal women				Postmenopausal women			
	25(OH) D deficient		25(OH) sufficient		25(OH) D deficient		25(OH) sufficient	
	*β*-coefficient	*p* value	*β*-coefficient	*p* value	*β*-coefficient	*p* value	*β*-coefficient	*p* value
FBG	−0.287	0.2894	−0.314	0.0579	−0.813	<0.0001	−0.172	0.0024
HbA1c	−0.133	0.3303	−0.238	0.0195	−0.443	<0.0001	−0.091	0.037
Insulin	0.181	0.4221	−0.036	0.844	0.566	0.0008	0.116	0.6164
HOMA-*β*	3.996	0.1824	1.734	0.3604	−1.464	0.506	−2.053	0.3684
HOMA-IR	−0.014	0.6581	−0.022	0.4862	−0.029	0.207	0.038	0.2708

*p* < 0.05 was considered statistically significant. FBG: fasting blood glucose; HbA1c: glycated hemoglobin; HOMA-IR: Homeostasis Model Assessment-Insulin Resistance; HOMA-*β*: Homeostasis Model Assessment-*β*eta.

**Table 7:** Effect of sociodemographic lifestyle and anthropometric indices on the development of vitamin D deficiency among pre- and postmenopausal T2DM women.

Variables	cOR (95% CI)	*p* value	Age adjusted aOR (95% CI)	*p* value
Age				
39-40	1		1	
41–50	0.533 (0.098–2.895)	0.466	0.477 (0.087–2.607)	0.393
51–60	0.733 (0.108–4.992)	0.751	0.678 (0.099–4.634)	0.692
61–70	0.700 (0.127–3.848)	0.682	0.530 (0.092–3.040)	0.476
Employment status				
Employed	1		1	
Unemployed	1.573 (0.851–2.905)	0.148	1.612 (0.828–3.138)	0.160
Educational level				
Basic	1		1	
Illiterate	1.194 (0.105–13.622)	0.105	1.095 (0.094–12.800)	0.943
Secondary	0.812 (0.449–1.469)	0.449	0.840 (0.461–1.531)	0.570
Tertiary	2.387 (0.257–22.173)	0.257	2.236 (0.222–22.529)	0.495
Duration of DM				
<5 years	1		1	
>5 years	1.824 (0.942–3.531)	0.075	1.842 (0.926–3.664)	0.082
BMI				
Nonobese	1		1	
Obese	0.763 (0.417–1.397)	0.381	0.749 (0.408–1.376)	0.352
WHR				
Nonobese	1		1	
Obese	1.375 (0.581–3.253)	0.469	1.419 (0.594–3.392)	0.431
WHtR				
Nonobese	1		1	
Obese	1.340 (0.728–2.469)	0.347	1.336 (0.723–2.468)	0.355

Binary logistic analysis was performed to determine factor that could predict the development of vitamin D deficiency. CI: confidence interval; cOR: crude odds ratio; aOR: adjusted odds ratio; DM: diabetes mellitus; BMI: body mass index; WHR: waist to hip ratio; WHtR: waist to height ratio.
